# Supplementary material for: Human papillomavirus vaccination practices and perceptions among Ghanaian Healthcare Providers: A qualitative study based on multi-theory model
Source: PLoS One. 2020 Oct 16;15(10):e0240657. doi: 10.1371/journal.pone.0240657 (PMC7567370; doi:10.1371/journal.pone.0240657)
Supplement: S1 File — (DOCX) [file pone.0240657.s001.docx]

Focus group 1

Nine participants (Health Care Providers)

**A: Knowledge about HPV**

I: Can you tell us about HPV, have you ever heard of it and if you ever heard of it what would you tell us about it?

P2: I have heard about HPV virus as a type of virus that can cause cervical cancer and also vulvar ones. I also heard that it can be transmitted sexually. And also during childbirth, it can also be transmitted from the mother to the infant.

P1: To tie to what my sister said, it can also cause cancer in any part of the body. Whether that be your hand… any part of the body. It can also cause cancer and warts

P5: Just adding to what they said, she indicated that you can get it through sex you can get it from oral sex, or anal sex.

P9: It can also cause genital warts in any part of the body.

I: If anyone person has the virus, what are some of the problems the person may have?

P3: It can cause cancer of the anus, that is the HPV

P5: It can cause cervical cancer.

P2: It can cause vulvar warts.

P6: It can also cause oral cancer.

P1: It can also cause stigmatization if it happens at a visible point in your body.

P8: Vulvar cancer

I. What is widely acknowledged about HPV.

P3: It can affect the genital area.

**B: Knowledge about HPV vaccinations**

I: We are now considering the management components it. As the health care provider, can you please tell us what you have been doing about what you know about HPV vaccination. Have already been doing about the vaccinations.

P9: The vaccination is called gardasil and cervarix. And it is given 3 doses or the recommendation is that 3 doses should be given. The first dose is given within the first month and the second month you will be given the next dose and six months from the first dose then you will be given the third dose. That is how they do it.

I: So who should be vaccinated?

P6: Ladies or girls between the ages of 11 and 12 are most advised to get the vaccinations, so at their adolescent age.

P9: Anybody who is trusted and is negative can be vaccinated.

P7: People with immunocompromising conditions like people living with HIV.

I: Yes and the age aspect like someone mentioned from ages 11 to 12 upwards.

P: The age I think it can start from 9- So I think the age from 9 to 12.

I: Why are HPV vaccines important?

P2: I think it’s important because it protects for a lifetime. If you are being vaccinated with three doses, it means you are protected from the HPV virus for a lifetime.

P3: It prevents infection.

I: Has anyone of you ever educated or counselled someone over the need of the vaccination of the HPV?

P9: Yes

I: If yes, how do you normally conduct such an education? How did you if you go about it? Is it an informal or formal approach or whatever?

P4: Informal.

P4: It’s like a conversation. Just let the person know the adverse effects of cervical cancer and give the person and education of what the HPV virus can do. So most often I use an informal approach.

P7: I also do it as an informal conversation because I just mostly talk about preventive measures seeing your not vaccinated. And it prevents you from sexual intercourse without protection. Yes so I will most likely do it informal to safe sex to help prevent them from most of these cancerous diseases.

I: If I may ask, as a follow up to what these people said, when somebody is resistant to the suggestions you are giving to the person, how do you go about handling the issue?

P3: I do show them the visual through the internet to tell you the effects of it if he or she does not allow us to talk to them.

**C. Initiation**

I: If you meet someone, how do you initiate, if someone comes to the health center and asks on this issue on different illness? How would you initiate or chip in for the person to be educated on this HPV vaccine?

P9: For instance, those of us at the family planning center, when someone comes there, we normally do education health education for them. So through the education or counseling we go beyond our counseling and do the cervical counseling with our client. We talk about it as the virus, how it occurs and how it causes cervical cancer and then the pap smear as well. So that is what we normally do there.

I: So do you normally as a normal procedure do you always include it or when somebody brings it up that is only when you talk about it?

P9: No. In the morning we do health education for all the clients so we talk about it to all of them. So it is a form of health education that we normally talk about.

Follow-up question: So is it being done as a preventive measure or as a treatment measure?

P9: The pap smear will let the person go and get the vaccination, because if the person is sexually active then they have to be tested before the vaccine. If they tested positive, they don’t get vaccination

So as a preventive measure

**D. Advantages and Disadvantages of HPV vaccination**

I: What are the advantages or why would you recommend somebody to take the HPV vaccination? If you take it, what are the benefits. Because based on the risk factors what make them be convinced to take the vaccination.

P4: As we said earlier on about the prevention, so after you take the vaccination, then you are prevented from having such infections.

P1: From a social scientist point of view, the vaccination, as you take it, it prevents you rather than to wait for you to acquire the disease and then start treatment which is expensive.

P6: I also think as said prevention is better than cure you need to protect yourself from these things. And I also know that every active boy or girl is prone to HPV so when you are vaccinated you are protected..

**E. Behavioral Confidence**

I: So how confident are you in believing such information to parents?

P4: The level of confidence is that at least I know the drug is very efficient and it works very well and so I have confidence in the vaccine

P3: And also other people have come for the vaccine and it has worked very well for them. So that will give much confidence in delivering any information about the vaccines to them.

**Follow up question:** So in an environment where people have stigmatized and they don’t want to hear about it, that talking about confidence, do you feel like you are well prepared to educate people in terms of addressing some of these people on stigmatization and getting people ready to go for the vaccination? And how are some of the ways you address some of the issues some people try to raise up?

P1: Everything is based on evidence and I can cite an issue of whereby there is no evidence that the vaccine gives complications or any thing. So it is based on evidence. Even though many of them have been administered, you haven't heard of any complications coming out of that so that confidence level is high and the vaccine is effective.

P; Also the origin of the vaccine, if you know the actual source of the vaccines, it would also boost the level of confidence to give it to client

**F: Physical Environment**

I: So the physical environment within which you will conduct such as a study, what types of support in terms of facilities and others do you need that will help you to offer counseling on people about the vaccination?

P9: We need Facilities, personnel, monetary incentives, education and refresher courses. We need the human personnel before you can initiate any move. Without human personnel, you cannot build the facilities and the whatever. If you don’t have human personnel, it would be very difficult to champion any course.

I: Can you be specific in terms of facilities?

P3: We need the human personnel before you can initiate any move. Without the human personnel, you cannot build the facilities and the whatever. If you don’t have the human personnel, it would be very difficult to champion any course.

Follow up: Can you help us, talking about human personnel, can you give us a rough idea about the number of physicians or caretakers that have been taken care of these kind of issues in the general population? In a rough idea, do you think that we have - I know that you said we need, that supposes that it is not adequate but I just want to see if there are any numbers that we can put to it.

P: No. It would depend on the target of people. The number of people you are targeting. That would tell you the number of personnel we would need.

P4: I think the monetary incentive is the most important thing. If you have the money, whatever facility you need, you will get it. Whatever technology you want, you will get it. So I think the most important thing is the monetary aspect of it.

I: You are health providers, you have been trained in the profession to deliver health care to patients do you need extra training in order to handle this vaccination as per the WHO standards?

P2 I personally i think we need additional training

All p: second we need additional training

P: need refresher courses for everything so that we get updated

P1: I also think whether we need training or not it depends on where the training is coming from. If it is the primary healthcare center then the training is definitely needed but if it is tertiary health center like komfo anokye, where we have specialized unit like oncology unit, i don’t think the training will be needed because they knowledge already so it depends on where the training is needed

P: The concern is about educating the masses to accept and patronize in the vaccination

P: new research will be done and new studies will come out so we need to get updated.

I: is there any physical incentives that you may require in order to implement or educate the people about hpv vaccinations, physical incentives?

All: of course physical incentives are needed (money, kits or tools, education) it will motivate us

**G. Change in social environment**

I: what kind of social support do you need to give the vaccination?

P: social support

I: that means support outside the hospital

P1: yes we need in the community and some of these could be needed under the care of parents, and we need ethical clearance or sometimes we need to explain it parents to understand before they can ask their wards to come or bring their wards so I would say the social support that we need is parental contribution or support. And if we are doing it in the community we need to get one or 2 opinion leaders and explain for them to understand the issue. So if we receive support from the community leaders it will help boost our morale to continue to offer education but before we receive those opinion leaders support they need to understand what the vaccination is all about. If community leaders provide a platform by mobilizing the people for us to provide education to the masses.

I: Tell us any social belief system in our environment, which may affect the implementation of the vaccination.

P6: Many people have this myth, I don’t know whether it is a something that have been researched into, that when you are vaccinated, for the ladies it will affect your fertility or your ability to produce.

P1: In akan, we say that “biribi anko ka papa engye trwerede” (literally translated as “if nothing happens to a palm tree, it will not make any noise) In other words, you don’t have any problem or at least to the best of your knowledge you are in good health so why need vaccination. sometimes you go for the vaccination and it will bring all kinds of problems. So why don’t I protect my health so I don’t go for any vaccination. Once the chance that you are going for the vaccination, you are calling for that condition to come to your system. We have that belief and I have experienced that before.

P3: I think there is a precedent, we have one nationwide vaccination for yellow fever we all contracted fever … malaria

P: So the general perception is if I’m not sick, what do I need a vaccine for? So they have this erroneous impression about vaccination.

P: Preventive measures are not part of our culture because we want to wait until something happens to us before we will go for treatment. If I am not sick, why do I need to receive vaccination. Others would say I don’t even want to know my health status to know kind of the disease I have. Ignorance of my health status is bliss. So based on our culture we have misunderstanding of preventive medicine.

P2: The fear of stigmatization is a social factor that contributes to people’s reluctant to receiving the vaccination. People want to stay away from STI so they think it will be a shame for people to know you receive HPV vaccine because they will think you have the virus.

I: What do you think about people's views that when you talk about HPV vaccination, to them, you are promoting promiscuity in your adolescent child?

P1: There is always this belief among the people when they know that they are immune to an issue, especially even our leaders who are in power, they are immune to some issues so they take a certain position. So once people are vaccinated they think that now I have my vaccination and I can go in and I can do whatever I want to do and it will not affect me. So when people are vaccinated, they are likely to go into sexual acts anyhow.

P: But I also think it depends on the counseling given to the person, if you are told you are protected don’t go about doing whatever, the person won’t do that. But if you tell them that you are protected for the rest of your life, definitely they would go about engaging in those immoral behaviors.

P2: I am going to add something to what my brother said, but it depends on how the counselor goes about the counsel because if you are being vaccinated, it only protects from HPV but there are other infections you can also have.

**H: Sustenance**

I: So let’s assume that you initiated the vaccination, what precautions would you put in place in order to alert others to tell them that the next vaccination is due on this date so child one or child two you should go for it.

P9: After I have given the injection, I will take the parents phone number so that because when it is about one week before the next injection, I will give them a call, so that is what I’ll do in order to remind them that their child is due for their next injection.

Follow up: So is that what you have been doing or is that the intention?

P9: No we do that. And them we give them a small book to write the next date for injection in it.

I: But in the meantime, if were assigned to implement this, would you need to change so you have time to do it? So if you accept this, will you be required to make changes in order to make this change?If you are supposed to provide education giving the vaccination will it be an additional task to your current task. Will you need to make any changes to your current situation or will you maintain and add it to your current tasks?

Most said they could still maintain with their tasks

P9: We can still maintain it.

I: Without any significant change?

P6: Yes we could do that without any significant change.

P: What change?

I: Your work schedule. You go to work from 8 to 4 or- But if you are assigned to incorporate this vaccination into your duty, and indicate-

P3: There is always a way to accept new opportunities and challenges and you always find a different way to solving some of these things. There is always a way to overcome some of these things.

P7: I think there can be change because if let’s say you are in a shift system and you are supposed to undergo these procedures in the afternoon or night shifts and you do this in the morning, definitely there should be changes for you to handle such a situation.

I: How will you overcome the situation that you assign the parents the adolescent to educate. How will you make sure that you will remind them to come for the next vaccination. I should be able to contact mother a,b,c, the next day this number of children, the next day a certain number of children, how will you cope with the situation?

P3: You create a proper timetable for all your plans .

P1: I think with the timetable, you can paste it so that , you can even set an alarm on your phone so that when it is due, you will be reminded of it.

I: Sometimes we attempt to educate the people and they may have a negative perception. What measures will you put in place to address those negative perceptions

P3: Sometimes the best approach is you get someone they respecte most or the significant others in their life to win them. .

P6: Like their chief, their imams, like their elders, their pastors, their family and parents etc.

P6: First of all I would let them know of the effects of them contracting the disease if they are not vaccinated. After that I will tell them of the benefits of being vaccinated some period of years after the vaccination.

I: What are some of the challenges you will encounter in the community about promoting HPV vaccination

P3: Resistance, people will not come out.

I: I don”t know whether you or you have ever seen or received the information of the malaria. If you happened to meet such challenge what will be the next action to do away with?

P9: As our brother said, you have to educate them for them to understand what they are going to do. If they only understand it I think the resistance will reduce. So you have to educate them very well to get their mind to it, they will understand it so that when they are going to receive it, it won’t be a problem for them.

P7: Most of these resistants are coming from people in the rural areas so you can talk to the chief and the chief will summon the entire group, the people in the village. So because of your in depth knowledge, you can lead the chief and they can help you in convincing the people in the village to accept the vaccine.

Follow up: So if yesterday or so three days ago I was listening to one of the prescriptions and somebody was so loud and ignorantly clear about the malaria vaccination. We as healthcare providers, how loud can we be to counteract some of these people who have these negative views and the way this guy was he sounded so convincing that I was afraid that our illiterate parents may hear this and they will be discouraged and they will be convinced. So as knowledgeable people like us what can we do more to counteract some of these louder people.

P6: I think most healthcare facilities around Ghana should create the awareness in the form of outreach programs, to educate the people. I’m sure most people are not aware of HPV so somebody can go to the radio and spread wrong information. But qualified personnel or people with knowledge about the vaccination should go on the air, either radio stations or TV to give correct information about the vaccination so that people will know about it.

P5: With regards to people who get access to the radio stations to speak to the nation, I think as healthcare providers we have not been able to convince the radio stations to appreciate what we have to offer so any “chap” just gets to a radio station and provide misleading information to the people. I think it is about time that the healthcare providers, got to this media house and let them appreciate what these concerns are so that when others come on air and offer any information people can judge for themselves..

P8:We at the family planning center. We have been going on air to educate our clients sometimes on the radio station to educate people. I have been doing that. And what we have been doing there apart from the family planning we do there, we also talk about cervical cancer. So I think when we get there we have to do it more as my brother was saying so that we can convince those listening to us.

P2: I think going on FM stations they pay before they can have access to those stations so if you are a healthcare professional and you don’t agree with what they are saying what it means is that you have to use your own money to go to the radio station before you are given a platform to educate the people. So that goes back to the issue we were talking about earlier on with regards to resources. So the first one we talked about was the monetary aspect of it, we also need physical things like the vaccination kits or the health insurance for the child or the patient.

P6: I think that NGOs should help make the vaccinations available to those who cannot afford it will have the opportunity to be vaccinated.

I: We are on sustenance. Having discussed all these issues, in terms of starting from education, actual vaccination, challenges to overcome. How can we sustain this program? So once you have given the vaccine to the adolescent the first time, how will you convince the person that they should come back and complete the entire dosage?

P3: Through constant monitoring by giving the person phone calls.

P1: You must also let the person be aware that incomplete vaccination makes the person still liable for the condition so I will educate the person to come and receive the three doses recommended to receive the full benefit of the vaccination.

I: Yes. That is generally. But at individual levels, if a girl has been vaccinated at your unit. How will you encourage the girl , the specific child, that when its due, day 1, day 2, day 3,the person should come for the next vaccination, how will you ensure that the person will voluntarily come for the subsequent vaccinations?

P2: Constant reminders by using text messages and phone calls.

P3: I will constantly remind them health implications of not receiving the recommended doses.

Follow up questions: How will you know your patient has the resources to come back? The person coming back to you, it’s not for free. And the person doesn’t have resources.

P6: Going back to what we said about some of the things that there should monetary funds that help them get to any appointment because they need to come.

P5: So we should make it available to the district and help those people to take off…

P: .Its not for free. So the moment the person doesn’t have the money to pay, can they get that education to come in…

P9: At the family planning, a lot of people come for the pap smear but they don’t go for the vaccination because they can’t pay for the vaccination. If they come and they are tested negative, when you tell them about the vaccine, they just go away and never go to receive the vaccination. Because the pap smear is 41 Ghana cedis, and the person is going to spend about 800 ghana cedis for the vaccine so they won’t go because they cannot afford it.

P4: I would suggest that the Government or the Ministry of health should take this as a serious matter and then collaborate with donor organizations to provide the vaccination for free.

P7: Apart from the government, you can also speak to some of these multi-national companies because as part of corporate social responsibility so that, they also can help us.

I: As a nation what are some of the challenges that we can come across with the implementation of the vaccination?

P2: Just as one of us here made mention of misinformation, audio circulation, such information can affect the knowledge we are going to impact on them because if, for instance, when I first heard of the malaria vaccination, I heard it from a lady. And that lady said it was a trial vaccination and we were going to be tested. But for this one I was coming and I was like is there any trust to this because she said some money needs to be paid and they don’t want to pay the money so if you are not so much into it and you don’t know much about it and you hear this for the first time, you will certainly be convinced so I think we should clear the air of misconceptions.

Follow up: So I think the same misconception we are talking about, even on the same radio, the guy was saying that the white people and those organizations that are dominating all those things for free, they are taking the whole nation ransom and trying the vaccination to see its effectiveness, and then they will take it away. These misconceptions are some of the challenges that as healthcare providers we need to rise up to the occasion to address some of these things because I heard clearly that we need philanthropy, we need organizations, we need all those people to come in, but when all these people are coming in with their dollars to support, here are a group of people who are resisting all those people are coming in to help. I think we need to do something about that.

I: Social components. What are some of the social challenges

P6: As I said early on, people have the mindset that when you are vaccinated, it will affect your fertility, so I think there should be more education about the effectiveness of the drug. What is going to happen to them when they are vaccinated. I think there should be more education on that that there is nothing bad getting the vaccination and these are the things you will go through if you are going to get the vaccination.

I: If we are going to add physical barriers.

P9: Can I add monetary problems.

P9: Because the vaccine is a little bit expensive.

I: Because you mentioned that in order to pay for transport to the vaccination, you cannot so how much more is the challenge. That is good. The physical barriers how do you-

P1: Some areas are very hard to reach. So you how to get the vaccinations there is very difficult. If I’m wrong, I don’t know about how the vaccination is stored, if they are the type that need to be stored in a very cold environment I don’t know. And those areas some are not even connected to the national grid of electricity so how do they store those vaccines.

The distribution of health care services across the nation is also very poor, sometimes it is only one nurse who is taking care of the whole community over 6 thousand population so that is also a challenge

P9: Can I add shortage of the vaccine itself. Sometimes the problem that we are facing in the family planning now, people come in to do the pap smear but we don’t have the kits for seven months to almost a year, we don’t have the kits. Look at this. We don’t have the kits for them to test whether they are negative or positive before they go for the vaccine.

P: So if someone is due for the next dose on that date what do you do.

P9 The pap smear will let the person go and get the vaccination

Follow up: So you are saying that you do the pap smear before they go for the vaccine?

P: Because if the person is sexually active then they have to be tested before the vaccine.

Follow up: So if we are talking about adolescents between the ages of 9 and 13, I believe you don’t do the pap smear on those kids.

P9 and 2: No. If the person is a virgin we don’t do the pap smear for that child. We do pap smear only the person is sexually active and even if the person is an adolescent.

P: How do you know if the person is a virgin or no

P9: We have to do the counselling well.

P: But some might not tell the truth.

P9: It depends on your counselling.

I: There some caregivers attitude, that may prevent the child to receive the vaccine. The child may be willing but the caregiver is preventing the child to receive the vaccine what will you do.

In other word, some of the feedback you get from the parents, when you suggest vaccinating their children, what are some of their reactions and comments that you hear from parents.

P9: As of now, the cost involved is very high, so when we suggest they will say as for the vaccine I can’t afford. So that is also something-

P: Generally all vaccinations come in the form of an injection. A lot of parents have a problem with injecting something into their child. Previously there was this Polio thing that when you give to children parent complain that their child will have problems. So if a company can adapt other modes of vaccination that will be fine, say oral or put in their mouth or something and ignore the injection because these are issues. Parents refuse to subject their kids to injections and all that because sometimes they don’t even trust the doctor to even give the injection. So going forward I think we need to find new ways to encourage vaccinations.

P9: That is for those who can afford. The rich people don’t want their kids to be vaccinated

P6: I think it depends on the efficacy of the vaccination. If parents are not aware of the efficient the vaccination is they will restrict their kids from getting vaccinated. So they should be told about it.

I: How about a parent who does not have time, they go to work and don’t come back home early, will they be able to take the child to go for the vaccination? When it is time for the child is to go for the vaccination but the parents are not home to tell the child to go for the vaccination do you think this will help promote the vaccination?

P9:. We health worker should put it on ourselves that we will track them when it is time for them to come we will alert them.

I: In Ghana we gave the government, the policies and the restrictions considering healthcare. How does some of these government policies that may affect the vaccination?

P: Positively or negatively?

I: Positively. Some of the policies that- If you look at it from both directions, what are some of the policies that are hindering it and what are some that are promoting?

P1: One policy is that we are moving from curative to preventive. So now we are testing all hospitals to formally solidify their health promotion and health education centers so that is a government policy that is going to strengthen vaccinations.

P: A negative one has to do with education. You get there and say we are doing vaccinations. This malaria one does this. So once you get there you didn’t get any knowledge on the malaria vaccination. So I think that our whatever ministry that is responsible for this education should always take it personally. It will let them know the benefits of the vaccine.

I: So if I get you well, there is a policy that will ensure that the people will be vaccinated and the other side of the policy is that though they should require that people should be vaccinated? But it will put in force and designate measures to ensure that each and everyone is vaccinated. Is that so?

P7: If the government can subsidize the amount you owe for the vaccination, or better still say it’s for free. So make an announcement that the vaccination for HPV is for free.

Daddy: So in terms of policies that we are talking about, do you think that government policies will be supported by data? Is it supported by data in terms of the number of people who have HPV virus, do we know the .. of HPV virus? What do you think about that as healthcare providers in terms of data driven to support the policies?

P1: HPV I would say we don’t know much or we don’t have much data on it. If we do it’s silent. It’s not like malaria or tuberculosis whereby we can just click and get it. So I am thinking government policies, in one way or another should be backed by data; but I am not very much aware of that of HPV.

Follow-up: You don’t know if we even do have existing data. And how about the vaccination, do we know the number of people vaccinated each year?

P: We don’t know.

P9: But I think the amount is not impressive because-

Follow up: On average-

P2: Oh its low average, 200 per shot.

P9: 600 per shot.

Follow up: So from your own estimation, on a daily basis how many people come for the injection?

P9: They don’t even come for the injection at all. Because we are the family planning center and when they are tested negative we send them somewhere else for the injection. But we don’t even have the kit to do the injection.

I: Right. So we have some of our cultural beliefs, rituals, ect., which may promote vaccination of the HPV and which may hinder? Quickly let’s consider some of our beliefs that may promote- what are some of the cultural, heritage, religious whatever that may ensure that people will be vaccinated against HPV? You know yesterday when we went to the family planning center, you said she was going for the counseling educational to go to church to promote education

P6: Most of the churches in Ghana, we have a health service after Sunday after mass or a church service, our head profession, maybe in 15 to 30 minutes or any issue. So for HPV somebody can come and we will tell them the good about vaccinations. But I know muslims are, most muslims viewpoint disagree with vaccinations. So some of these viewpoints can hinder the vaccination.

I: And how about our culture, cultural beliefs. And sometimes they use this public information center. I know! And it’s allowed. Getting the people their health insurance cost to be renewed, a whole lot. Do you think that whenever we channel the vaccination program, through these channels it can register positive impact?

P1: It can register very positive impact. In earlier years, before we are aware we are bringing other issues, other health related issues.

I:So what are some of the negative things to our culture, beliefs, rituals, ethical, social environment, what are some of the negative aspects that may compete the dementation of the vaccination.

P2: I think refusal to involve the chiefs in these activities because those who are responsible for rural area, thay have belief in their chief, so if we bother them and we do our own thing, they can also give them permission that they cannot listen to us. So I think if we involve them it will help us.

I: I don’t know is some of you have ever… if you fail to receive permission from the chief priest, no one will except you in his or her house. No one. So the system to recognize the goal of the leadership, it helps the dementation. Think of other limitation factors because we are running out of time. If someone vaccinated three. Four years ago,-

P6:... that when you are vaccinated, this is what will happen to you. But I think that there is more of a reason to clear that perception or mentality. It’s all about the misconception. For instance. If someone goes for vaccination and dies, they can relate that that vaccination killed the person.

P8: We should educate the community well because sometimes they will- sometimes when someone gets a disease they will tin its eduabum, so we have to ediate the community well and they will accept it.

I: Last question… I think you have heard vaccination programs whether its community based or we gather people based on where they are such as ate a mothers day or whatever and they come there to be vaccinated; whether it is community based in their homes or hospital based, which of these programs do you think will be more effective.

P1: I think door to door will be better because if the person is their for you to vaccinate, they are not going to say anything, the person is there they don’t need transportation. You come there, educate the person and give them the vaccine.

P9: I don’t understand what you are saying because you can’t just vaccinate someone without doing the pap smear first, if you go to the door of the person and just vaccinate, how are you going to make sure that the person is positive or negative. They need to be tested first before the vaccine and if they are positive we don’t give them the vaccine.

P9: With 11 to 13 you still have to do the test to be sure that they are still a virgin before you vaccinate the person. So you can’t just get to their doorstep and just vaccinate.

P6: In order to ad to what my brother is saying, you know, some of us Ghanians, we want the vaccination. But we don’t want others to know we went for the vaccinations. They like the vaccination but they don’t want others to know they went for it.

I: Some times people know they engage in sexual things. But sometimes they are scared or they don’t want anyone to know or whatever it is. So you to establish the education and discover the truth before you give the vaccination right.

P9: You can give it to a positive person…

**Focus group 2**

**A. Knowledge and awareness about HPV**

I: How well do you know HPV?

P2: I know it’s an STD

P1 It’s supposed to be contracted through sexual contact

P3: i know it is associated with warts

P7: I know it can cause cancer, its human papillomavirus

I: If someone has HPV what possible health problem will the person likely to have?

P1: The person can have genital warts. The person can also have cancer

I: What type of cancer are we talking about here?

P1: cervical cancer, neck, throat, anal etc

PI: I think it is transmissible, as one has it and has unprotected sexual contact they can transmit to somebody else.

I: how common is HPV?

P4: I think it is between 2 and 4% prevalent in this region

I: Do we have any data to support?

P4: i think there was a study that was done at Ashanti Akyem Agogo and that study points to the prevalent being around 2 to 4%. I think there was a study like that.

I: Can you tell us the reasons why HPV rate is low like that, is it just because people are not being tested for it or is it because we are not keeping proper data?

P7: one think most people don’t have the education, we don’t have knowledge of it so you will not seek help. They may take it as normal disease and some may even end up at prayer camp, herbalist and so on.

P5: We don’t have data specifically on HPV. I think once it is viral, it mimics characteristics of any other viral so I think we just lump them together and reporting is a problem so if education goes up and the reporting patterns changes then we can report on it really

P4: In the same line just what the number 5 said, yes, I also support, few studies have been done in this area and the other side is that people are not actually looking for it to quantify the disease burden.

P2: there is no awareness, if people are aware and they report the problem, it will come out

**B: Knowledge about HPV vaccination**

I: Who should be vaccinated?

P1: Every woman who is sexually active or every young girl who is growing up should be vaccinated to prevent it. It is not only who are sexually active in some way you might get it in another way or another form which are probably they may have a small percentage so somehow they might be vaccinated, everybody must be vaccinated.

P5: I am adding to what she said, everybody but she mentioned young girls. I don’t know whether it got nothing to do with men, I don’t know so. The focus should be on the youth, when you analyze sexual activeness with age categorization, you will see that youth dominates so if the vaccination can target the youth I think it will be proper.

P8: I think it is not only the women or ladies who should be vaccinated, it should include everybody so that it will not be transmitted

I: At what age should one be vaccinated?

P5: As for vaccination, if we can probably target as from 12 up to 40 I think it will help a lot had it not been that, like I would say we have to even screen at birth as being done for sickle cell so that we prevent it entirely.

P3: Also think it should start from age 9, now the adolescent age, we talk about adolescent age is reducing, last on the news I heard about nine years are getting pregnant. So i think it should come down to nine.

P8: I think anyone who is below 18 cannot consent or agree by law unless the person is 18

P5: I disagree, in most studies the parent will come for the consent of their wards, so those under 18, if their parents are there or their guardians are there, they can step in to fill the consent.

P4: i support the initial concept that people could be vaccinated at birth, in that way once you are born and you have immunity to the HPV. At birth you have the vaccination to protect you.

P3: Adding to what he said, because of social manners like rape and others, I think they should also go to age zero

I: How many doses should be given to people who want to be vaccinated?

P5: I think it is dependent upon the person’s age because if you are giving doses to an adult the same quantity of doses cannot be given to children so I think depending on your age then some doses may be recommended to you.

P4: I also think that for hpv virus, pharmacokinetic condition studies need to be done there is where we will be able to established actual dose that is required for the age we are looking for.

I: i need to clarify the dose we are talking about here. We are talking about the number of times the vaccinations have to be given to the individual.

P7: I think it should be given twice, I read some things sometime ago and they say you can give it 6 months interval. When you give the first dose, 6 months time you can give the second dose, depending on when the dose was given, let’s take it that the child is 11 yrs old now and the child qualifies, tested has been done and the childt is negative, and you give the vaccine now and you count the six month time and you give the second vaccination. I don’t know the dosage whether 5ml or 6ml, that one I don’t know.

P4: I also think that the number of times should be dependent on ones immunity. Because somebody could be vaccinated, let say today, and then the virus leaves the body system, who know, for number of years, you think this person has immunity to HPV but he may not so the virus comes, but let’s say periodically people are tested and once you are tested and you don’t have the immunity to the HPV then you give the vaccination to the person rather than given it every six months. That is what I think.

P6: I think the six month interval that the two doses needed for a person is a lifetime something we are talking about like what we give to our children bcd single dose for lifetime. You know of yellow fever also every 10 years you can also boost it. That two doses will be adequate for a lifetime immunity if one follows that interval.

P4: I disagree this is my reason for that one given the age bracket within which the HPV occurs, the age bracket, assuming let’s say the age bracket is 18 yrs and above is sexually active and then you give it let’s say at birth, now you’ve given it at birth and now you think this person may not be sexually active now you think that the spread of the disease may not be that much, So once the person becomes of age at 18 yrs, let’s say you give it at 18 and let’s say at six month interval, perhaps this person may be out of the system and he will lose immunity entirely, that is my ground for disagreement.

P6: I know that when you receive vaccines and the antibodies are formed you are protected for life. If the antibodies are formed anytime you come into contact with the specific organism, the antibodies will activate themselves so once you develop the immunity, you receive the number of doses you are required to get, it’s for life. After one year, if you give a child bcg and the scare is formed we anticipate that this child is protected for life. When you talk of vaccines, we are assured that when the antibodies are formed you are ok.

P7: The moment the vaccine is introduced, the body develops memory cells so when the vaccine is given the second time, it will boost it.

I: Have we ever talked to parents with an adolescent child about HPV?

P4: never

P1: NO

P:6: NO

P5: NOT AT ALL

P3: NO

I: What are the reasons for the nos?

P6: It’s one of the conditions that we said it is neglected, we don’t focus on it. Because we don’t have data and I don’t know what it is public health importance to authority. So once we don’t have interest in that aspect even education and awareness may not be done.

P4: I am not aware if hpv vaccine is available.

P7: It is not our priority. We have some diseases like malaria, HIV, TB that we focus on that. Like Ebola, those that can cause pandemic or epidemic, the rest but for HPV it’s not our priority so we even don’t talk about it.

P2: Yes, sometime I do. A friend of mine her daughter had a condition and I had to run around and get a doctor. It is uncomfortable so that when the teanagers come around you ask them have you experience anything like that before, like sometimes you have to examine them if they had it, I give them small education to take good care of themselves. There is something like hpv that they should take care of themselve.

P5: We are not talking this serious as HIV that is why are not sensitizing people about it.

I: how common it is for people to have cervical cancer, throat, anal, mouth or neck cancer? Is it because we don’t have the data to support the number of cases of people who have all these type of problems? Or is it because people are not tested to know their status that is why it is not prioritize or is it because cervical cancer is not common at all.

P1: For cervical cancer it is priority for ONG stuff that we always talk about, when you come to the OPD section, we always talk about cervical cancer, they symptom and how you can get it, much education have gone into cervical cancer because it is a lot in Komfo Anokye, because of that we created Gynecology clinic for it and every Monday, so every Monday many people come for cervical cancer treatment.

I: if cervical cancer is common as our sister is saying then we need to prioritize HPV vaccination because study have shown that cervical is caused by HPV and therefore the vaccination should be important

P5: I have some quiet dissenting view to what you just said, if HPV is the lead contributory factor or is the only way one can get Cervical cancer, then our data is not correct. Because there as aspect of our work, we are using the ICD data to code all the ailments that are reported here. So if the doctors catering for such cases lump everything up and write CL of the service, without giving us all the other contributing diagnosis or ailments that hover around it, then we are also not report on HPV. So data in African and Ghana for that matter is something.

P9: As it has been said because we created awareness by giving education and going into the community to talk about HIV, so we were able to gather data on it.In this case, that is, about HPV, the awareness, the education and data collection and community surveillance can also help use to get the people to talk about HPV and that may help.

**C. Advantages and Disadvantages of HPV vaccines**

I: What are the advantages of HPV vaccination?

P3: I think it will prevent you from getting the cancer we are talking about.

P5: Your antibodies will be develop and your immune system be stronger to fight the virus

P4: I also think that you only derive the maximum benefit not when you have had it so you will have the benefit of the vaccine not when you have had it but before you had actually introduced the vaccines into your system so you can boost your immune system.

P7: It think it will reduce the morbidity and mortality associated with the HPV

I: so to add to that if there are other advantages you can bring them out

I: what are the demerits of HPV vaccines?

P1: Every vaccines has its own way of making people uncomfortable or has it own side effects. It may make you feel weak.

P5: Some traditional beliefs will prevent some people from receiving the vaccine but in Ghana we are lucky proper education and other things, the merits are toned down so the merits outweigh the demerits and in ghana all the tribes all the community have come to agree to vaccination so we are lucky so I believe as for vaccination when traced its history very serious.

P6: We know that when you give vaccination to somebody, the person is protected for life, and so if I am vaccinated and I am assured that I am protected against that I think it will encourage people to engage in immoral activities. Like if we were to have a vaccine for HIV, you know because of HIV when people are engaging in immoral activities some time they are cautious because of HIV so I think that vaccination although is good, if you have a vaccination for this condition it will encourage immorality.

P1: I think in the US or UK when children are growing up you can talk about family planning but in Ghana you can’t practice family planning in Ghana because they might think that you are putting something in your child’s head to engage in immoral activities.

P4: I also think that when you give HPV vaccination to a child you are promoting a promiscuity

P7: The stigma attached to HPV, if somebody dies of Hepatitis B people will not make noise, though it is caused by STI. it depends on how you sell the thing. Last time we were doing YF (yellow fever) vaccinations, at one community. It was a muslim community and the people were not coming out to vaccinate and the Chief Imam was consulted and he came and vaccinated, that is all, the whole community came and vaccinated

P5: It means that the entry of that program was not planned well. If you know a community head is there and you want the followers to go by whatever that you are doing target the community head and everybody will follow.

**D: Behavioral Confidence**

I: What will make you confidence to decide to vaccinate a teenager with HPV vaccines? What will be your source of confidence, is it because of drug

P4: Efficacy of the vaccine will make me confidence

P5: He said it all, because the efficacy and the awareness, when people become aware of the dangers associated with HPV, with little effort they will be interested in it.

P2: The parent consent will make me feel confident. The involvement of the parent in the child’s health will give me confidence to give HPV vaccination.

P3: It think it should be encouraged from the national level where there will be education from the national level about the seriousness of the HPV and its health implications. Like HIV people are aware of its health implication because of the involvement of national leaders and awareness created by the national media. So if we show on the TV the problem associated with HPV and the problem with cancer, I think people will become aware. In most cases, people died of cervical cancer and they are not aware and the family member will be saying that the person was just sick and went to the hospital and just died but if we create awareness, people will see the seriousness of the problems.

P4: The safety profile of the vaccine will also make me confidence to give the vaccine to adolescent. Even though the vaccine may be efficacious but I will look at the safety aspect of the vaccine

P6: I think the availability of the vaccine and the facilities will make me confidence to give the vaccine. If the facilities are not there and the place is not well organized then it will not encourage us to give vaccination

P6: The research findings should also give us encouragement. The research findings should be disseminated well in languages that will be understandable or accepted by the masses. There are some research when you read the journal you see it to be like jargon, words that you have never met, but we want to use those research finding to help the population but some of the research language are difficult to bring it to the level of the people.

P3: The personnel is important. People who delivery the HPV education and the vaccines need to be trained well. Most cases, they used scare tactics and if care is not taken the strategy to communicate to the people will backfire therefore they need to be sensitive to the culture of the people. If the are trained well and are sensitive to the people, they would get a nice reception and be ready to receive the vaccination.

P6: I don’t know whether the vaccine is for sale or its for free, if it is not for free, how many people can afford. So if we want to reduce the morbidity rate, as we are talking about, then this vaccines ought to be free. If it cannot be free then it should be affordable so that many people can patronize.

P5: I want to look at if from that the government will buy the vaccines and make it available to the ordinary Ghanaians. The government can buy it, reduce the price or subsidize the price to let’s say 5 Ghana Cedis, [less than $1.00 equivalent] that will help others to go for the vaccinations. Simply put, there is no widespread, systematic, publicly available HPV vaccination program in Ghana to increase vaccination rates.

P9: affordability is the issue. Some of us we work in poor areas and if you look at the people and their situation, there is no way they would be able to afford it even at the cheapest prices and those are the people who need the vaccination the most.

P2: Sometimes you would like to give the vaccination and they would tell you the vaccine kits are not ready for you to be tested and you will be going up for about 6 months to a year and you would still not get the vaccine kits to give to the patients or to test the patients. So sometimes you just have to forget about it.

**E: Change in physical environment**

I: The physical challenges one may encounter when you decide to administer HPV vaccine to an adolescent.

P2: the availability of the vaccines. If I educate somebody and if the vaccine is not there what are we going to do? So the vaccines should be available at reasonable prices before we even talk about education.

P7: the adverse effects following immunization needs to be looked at. We need to information them about side effects, let’s say we need to inform them that after receiving the vaccination you will feel dizzy , you will feel nausea so that the fellow may be aware of the side effects. If you don’t do that and an unexpected happens they will say the vaccine is not good and it will discourage others from receiving. We have witnessed similar instances where a vaccination is given to somebody and after that something happened and the person dies they associated the death to the vaccination.

P4: We need to have access to information, how safe the drug is, the studies that have been done to evaluate the safety and efficacy aspect of the drugs to convince myself that it is safe and efficacious, that is when I will give it to others. So the information about safety and side effect should be transparent.

P: The information about the vaccine should be translated into the local languages so that people can read and understand the safety, side effects and the effectiveness of the vaccine.

P5: We also need to create a registry for HPV administration like we started cancer registry but at the end of the day so many things happened, i don’t want to go there, when we have a register it’s then that we can track when we started given what and what are the issues? Since people are going to receive the vaccine there should be a system to track those who have received the vaccine.

I: So are we saying that there is no registry for the hpv vaccination in ghana?

P5: No no, no. I don’t know but I can say for a fact that cancer oncology we were having a sort of registry which was serving a valuable people but I can say for a fact that HPV was not considered and we don’t have any registry for it.

P5: About the coding of the diagnosis to the registry, whatever that the doctor’s final diagnosis is, is what we code.

i:Does it mean that some of the diseases will be categorized under one code, for example neck cancer throat cancer, cervical cancer would be coded under one code?

P5:No, we have multiple coding. What I am saying is that the doctors may treat somebody with cancer of the cervix and on the balance of probability will weigh that this and that, I think was more pronounced than HPV so let me conclude by saying that it was the disease of the cervix and HPV will be ignored

P4: If we do not have HPV in the coding process, and we want to quantify the disease burden of HPV then certainly we will not be able to know the prevalence and incidence of the disease burden.

**F: Practice for change**

I: What are going to do different from what we have been doing to increase the awareness?

P2: I don’t know if the authority will allow, I suggest that they include the HPV screening into the regular screening requirement in the country so that every woman who is due for screening will be screened. So it should be mandatory

PI: Pap smear is not a routine, those who come to obg with viginal discharges or are sent to family planning center to do the pap smear those are the one that we will do the pap smear. Pap smear is done as a diagnosis for treatment but it is not done to detect whether a person has the HPV virus. It is done only when the person already has a problem.

P9: the awareness needs to be created so people who are aware will be going for screening.

PI: Some clinic did cervical cancer screening and myself, I went for it. So somehow because we are given education about cervical cancer some of us went for it.

P3: We should also make the HPV screening kits available to the clinics sometimes you go to the center and they will tell you we don’t have anything available for screening so we should try to make it available.

P5: If we can make it mandatory for all health workers to screen for HPV and the vaccines if it is available, we give it to those who qualify.

P4: We have ethical obligations to direct somebody, for instance, you are screening somebody for HPV and you should be able to tell the person to go for the vaccine, but the question is, is the vaccine available for the person? Even our locality here at this big hospital we don’t have the vaccine, so if I screen somebody and directing the person to go for vaccination and the vaccine is not available, then you ask yourself once you screen ethically are obliged to direct this person for treatment or to go for HPV vaccine of some sort. So stakeholders making the vaccine available to the market is the first step and that is when we can begin to educate the people about going for the vaccines.

P9: I suggest that we also need to train the personnel about the vaccinations.

I: What are the challenges we would encounter as a nation in introducing this HPV vaccination?

P9: Government needs to set up funds to support it.

P5: If HPV vaccination toes the lines of malaria vaccines were introduced to the system now then it is going to fail. Because there wasn’t enough education in relation to malaria vaccines. In fact, introducing this HPV vaccines, what we should do is to create the awareness and series of workshops for healthcare professionals and the communities and I think it will see the light of day.

P6: There are too many vaccines in the system, and so once we are to add and I know it is injection, right, they are people who are at 12 and 13 so adding on another vaccine for people to come will be be another issue

P4: Health workers should be convinced first and regardless of what other are saying because we are convinced we can educate the masses

**G: Social environment**

I: What social, political, religious factors may hinder or promote HPV vaccination?

If the institution support it

P2: I believe that in the case of vaccination, if it is done in communities, that is community by community, for instance in my community, let’s go and get the chiefs and the assembly men involved and the pastors involved talk to them and convince them. Most of us are christians and we have muslims. So if we convince ourselves we can talk to our pastors and our pastors can allow us to talk to the congregation about the vaccinations. If we involve our pastors and assemblymen and if the pastors and assemblymen take the platform to talk about the vaccination the people will be convinced. Involved the opinion leader involved in the education process.

P3: I think we should go through the politicians because people don’t trust politicians anymore. Because people think that politicians have been bribed and that is why the are asking us to be vaccinated. So it think we should go through the health workers, because most trust health workers like doctors more than the politicians.

P5: In fact, the last questionnaire about religious affiliation. If the youth got to know of advantageous, it may lead to more promiscuous life. It means that the message that we are going to send should be a message that will not promote promiscuity. It should be a message that will drum home healthy lifestyle and prolong life and good health other than that it will backfire from the blast of the whistle. I think we can rely on politicians because they wield power to influence people in their constituencies to participate in vaccination. We have to know how to enter a community, enter a church, enter a mosque, enter whatever area we want to go to educate.

P6: Good community entry will always get you the people you need.

I: What method could be used to get the community to accept vaccines

P2: Through the opinion or community leaders, the volunteer, the headmaster. You give something to the chiefs in the community or perform tradition rite within the community to get the chiefs on board the you can easily reach the rest of the community. You don’t go to the community empty handed. If you go there empty handed they will even sack you. The volunteer and the assemblymen can convince the people so we need to involve them.

P1: we also need to utilize the media. We also need to be an agent of change in my community. I always give education in my church about any health issue that may come up.

P5: I want to suggest the communication component of those who will be go around for education. Communication team, those who will hold radio programs, TV programs, go to churches and all serious gatherings, who can speak with empirical evidence will help to bring the message to bare about the vaccines.

P7: I side with number 5, when a clear message is not given then it will give a wrong signal and people will have misgivings about the program. A typical example is when we were doing yellow fever vaccination and we were talking about mosquito nets and measles and people misinterpreted the messages we were given to them that if you don’t sleep in the mosquito nets you will get measles, meanwhile, that is not what was said.

Focus group 3

**A: Knowledge about HPV**

I: How many of you have ever heard of HPV?

All of you said yes

I: It looks each and every one has heard of HPV. So can you tell us what you know about the virus; HPV. Briefly, can you tell us in a summarized way what the virus is? HPV. Some of the symptoms. Symptoms, do you know some of the symptoms?

P 2: Some of the symptoms are, the most common ones are the genital warts.

I: Right. Do you have anymore?

**B: Knowledge about HPV vaccinations**

I: Let me ask you, is it important to vaccinate people against HPV. Is it important?

P4: It is; It is because we are being told that when you are being infected by this virus, the likelihood of you getting cancer is high. So it is very important to get vaccinated to stay away from cancer and all the others.

I: Thank you very much. Within what age range do you recommend for the vaccination.

P1: So teenagers have become sexually active, so if we are to vaccinate, it’s better to start from the secondary schools, even possibly the junior secondary schools here.

I: Thank you so much. How many have you ever discussed the vaccination program with parents whenever they come to your consulting rooms? Have you ever?

All said no

I: So it’s not a topic that is commonly or frequently discussed in the hospital in general, right? If no, then why haven’t you discussed issues on HPV with your patients?

P3: I think it’s because of the clinical situation. You know when the patients come your aim is to address the condition they present with; so if it doesn’t go in that direction, it’s not likely that you bring that topic up.

I: Right. So why haven’t you discussed HPV issues with your patients?

P5: For me I would say because of the rotations I have been doing. I started with pediatrics so working with children. That is why I have not had the opportunity to go into it.

I: Right. Have you ever?- you haven’t, good.

**C. Initiation**

I: What are some ways in which you come- it has already been clear that you have not been discussing HPV virus issues with your patients- but if it happens that you should discuss, to brief your patients on the subject, how will you go about, how? How will you initiate discussing these issues with your patients?

P4: First I want to know their knowledge on cervical cancer because it’s the most common cancer caused by this virus. And if they have any knowledge about it I want to also know if they know the risk factors of getting this virus if they are sexualy active; and I want to tell them the benefits of the vaccine.

I: That is good. So what will be your approach.

P7: For those who are sexualy active, especially when i’m taking there are some conditions that will take your sexual history or your reproductive history. So I can use the opportunity to speak to those who are sexualy active about the virus, and then the various conditions that can be caused by the virus.

**D. Advantages of HPV vaccination**

I: Thank you so much. So now we are on the participatory dialogue. Some of the advantages- the reasons why you recommend the vaccination of the HPV to your patients. Or you will advise your community that they should go in for their vaccinations. What are some of the expected benefits that will prompt you to embark on promoting HPV vaccination to your clients, your patients, your community, whoever?

P8: I think cervical cancer is the second leading cause of death among cancers in women, so then I think the awareness has to be increased , so at your approach at trying to create more awareness, you’re supposed to get more people on board, especially the teenagers.

I : Right. Why would you encourage teenagers to go for the vaccinations?

P9: Just as I earlier mentioned, you know that when this virus is in contact with your system, you are most likely to get these cancers, so to avoid the cancers, it is better we get adolescents and even female adults to get vaccinated to be prevented from getting these cancers.

**Disadvantages of HPV vaccination**

I: We have discussed advantages and for every advantage there is a disadvantage. So what are some of the disadvantages that may arrive against HPV? What are some of the disadvantages, is there any disadvantage?

P3: Some females wouldn’t want their peers to know they are sexually takactive or they want to become sexually active. So in order not to let them know, they wouldn’t even go for it in the first place.

I: So going for it will expose them and their hidden agenda . So what are some of the disadvantages ?

P6: For the vaccination, they have to be injected so the pain caused some of them from getting the vaccination.

I: Right, good. So when you are getting the vaccination, are there any disadvantage or are there any pain or are there any discomfort of these that may prevent people from getting the vaccination.

P1: Not all patients like to be injected, in fact, the majority of patients would avoid injections as much as possible so I think that is one of the things that will deter them. So after the vaccination, it comes with so much pain , for example the … vaccination, it comes with so much pain that the arm will hurt for quite a number of days so discomfort, it will deter people from getting vaccinated.

**E. Behavioral Confidence**

I: Thank you, thank you. So what will motivate your level of confidence in giving the vaccination if you have employed, you have been assigned to coordinate the vaccination program. Your confident level, can you confidently accept that assignment and proceed to work as the requirement? Can you confidently accept the assignment? Yes or no?

P9: Yes

P10: Yes

P5: It will depending on the skills we have to execute that assignment. If the facility is renowned like ours people have so much confidence in us and so whenever something is coming out of kofomache they have so much trust and that will reach out to a lot of people.

I: So if I should ask you about your level of confidence, in terms of 1 to five where one is yes and the top, five, is weak, how will you rank your level of confidence? 1-2-3-4-5.

P3: ⅘

I: ⅘

P2:⅘

P1: ⅗

I: Good. good, so the facilities and what else? Skills available, necessary tools- logistics

**F: Physical Environment**

I: Good. Let's consider the environmental, physical environment. What are some of the physical environmental conditions that may require, that you need to meet before you can provide this vaccination- the services.

P3: So before you can provide these vaccines and vaccinations you need money; you need to be able to provide the needed tools and the needed logistics.

P4: Facilities

I: Yes. So how does facilities come in whenever you are embarking on vaccinations?

P5: I think in trying to reach a large number of people you need to have a convenient advantage so that people from all around can converge at that point to receive the services you are offering.

I: So you need a suitable place to operate, right? How about mobility? How does it come in?

P7: When you are able to get to these points of vaccination, you need to at least travel from one point to the other. Your means of transport one and also the available roads and then the other means to get to where you are going to do the vaccination is important because some people may be hindered because they have to travel a long dusty road and by the end of the day they will be- their clothes will be filled with dust and they will be tired; so they will not be encouraged to even attend even if you were to organize a vaccination.

I: Right, right, and even the current situation of our rules, how does it come in; or post challenge to a nationwide program?

P8: In terms of roads not motorable here, and in such cases, and it deters people from making long journeys or even making it to where these services are to be rendered; especially when you want to go to the district and offer these services, it will deter people from coming. One, the roads are not motorable so you don’t have buses or other means of transport through those roads, so it would be very difficult for people to make the journey to the point where the services are being offered.

I: Right. So what are some of the incentives you need to ensure a successful vaccination program?

P9: It would be very appropriate if we organized a means of transportation for your target group so that you know that we are taking care of him on his regard. We would also need to find some snacks and refreshments for them so that they know that when they come they wouldn’t have to spend their own money to feed themselves at that place before they go back home so these are some of the small things you can do.

P10: In other words, you will be required to motivate them to come. And you know when they say motivate us, It means that you would have to dash out some money to them to motivate them to come. So these things will also be required to carry out such-

P6: You would also have to motivate the staff that are involved with the vaccination because if you want to get more people involved, people to volunteer to do this,it would be good to motivate them in cash.

**G. Change in social environment**

I: Thank you so much. Then, we are considering social environment- personal relationship, the tribes, whatever. What kind of social support you may need whenever you are embarking on vaccination on HPV?

P2: Involve the community leaders because once you get there, there are some and they will know how best to communicate with their subjects. They know how to communicate in order to get them on board. So once you are able to get the community leaders and then dispel all myths and they are able to adequately convince them on the importance of the vaccination, then we will be able to get more people on board.

P7: In our communities, we hold the churches in high esteem so to involve them to be able to advocate for the vaccine would be an advantage for the program.

I: Is there any … by your traditional leader in our social settings?

P4: Yes. You see for us, we also hold our chiefs and community leaders in high esteem. In some communities for instance, you cannot just enter and talk to anyone, in the more muslim communities, you will be required to get the chief involved before you can be accepted into the community, so chiefs are so much important to such an excersize.

I: Thank you. Ant to what extent will social practice prevent olr serve as a challenge to a vaccination program for HPV?

P4: Talking about churches, most churches will not encourage pre-marital sex. So they may see it as a means of promoting immorality when you vaccinate adolescents and teenagers, they are seeing you as amoral and they will not support it because of that.

P6: Some communities also frown on vaccinations because I understand in Nigeria a community in Nigeria does not take vaccinations at all. So we have to dispel these rumors before we can carry out this kind of exercise.

I: Right.

P5: For most of these children, some of these will be below age range so their parents approval too is very key to be able to carry out this vaccination; so I think when parents are not on board, it will be difficult to just go vaccinate someone his or her parents approval. Sometimes it would be good to create awareness even as we create awareness in our schools, we should also try to educate parents more on the need for such a vaccine so that they will be able to release their wards to be able to go for these vaccines.

I; Thank you. Let's assume you started a program and participants have participated fully and they are supposed to come on two or three occasions. You vaccinated them on the first day and they are supposed to come on the second and third occasions. How will you encourage the participants to attend the next vaccination page?

P8: So aside from speaking on the need for the subsequent vaccination, I would also want to maybe get cards for them that will register or record these dates for vaccination. That is more practical. And also follow up on the by means of calling them or sending them a personnel to their homes to remind them of their next visits for their vaccination.

I: Good. Will you be willing to get them aware of who they are supposed to come to for their vaccination?

P9: In this more technological age, we want to- if they have a phone, when you want to help the person, input it in their alarm system so that maybe two days or a day prior to the vaccination the person will be reminded.

I: Good. How will you encourage the parents to know that on this date at this time they are required to come for the vaccination? What will you do?

P10: Practically the best you can do is- I don’t know if it’s a community, you can go around and make an announcement because sometimes they have the vans that go around or on the community leaders terms, you can regularly make an announcement, especially if its a week in time or two days to time you could go and make an announcement there.

I: Thank you. So whenever we mention vaccination, what comes to your mind?

P1: Protecting you against a preventable disease by introducing a dead or weakened pathogen into your system.

I: That is good. What are some of the advantages- it has already been mentioned but I want you to repeat, what are some of the advantages of being vaccinated?

P2: To protect you from a disease that is preventable. It is also to, you know it protects the community depending on the number of people who are vaccinated, even if someone comes into the system with the disease, you know it only takes others who are not vaccinated. So it is also an advantage.

I: Good. So in respect of the vaccination, what are some of the negative feelings that will come to your mind regarding vaccinating teenager, or adolescent girls?

P3: The vaccination sometimes comes with diverse reactions you could have pain at the sides, you could have an allergic reaction or a sensitive reaction to the vaccine.

P4: I also think that peer pressure can influence the girls who we need to make them aware of the benefits so that even if they hear bad things that could happen, the would rather come to the health professionals advice instead of peers.

P10: You see, the stigma that comes with it, especially the things that- there is this notion that once you go for this vaccine it means that you have the intention of either engaging in sexual activities or already doing so, people stay away because of the stigma associated with it.

I: Thank you. You have given us a number of disadvantages. So as physicians and whatever, what can we do to overcome these challenges? How will you address these challenges if you are trying to go for a nation vaccination program? And these advantages that have come up, how will you address these over the disadvantages?

P5: Advocacy

P6: So periodic reminders on the advantages of the vaccination, so from time to time, just remind them of the advantages.

I: That is good because if someone is convinced that if someone knows the advantages in respective of the disadvantages, it will still go.

P7: Not to scare them, but you can also let them see the terminal stages of people with cervical cancer and then it will make them know the severity of this condition so they will know that going in for this vaccination will be preventing them from something that they wouldn’t want to experience.

I: You mentioned something on the program and according to the instructions on the guidelines, you are required to issue the first dose second dose and third dose. How will ensure that people comply with the subsequent doses.

P8: First I think the organization should be well done, there shouldn’t be lapses or maybe the people will come and those organizing the program are not ready, it sends a wrong signal that you are not serious about it. Secondly, if incentives are provided, the first time they come, their incentive because they know that if they come, there will be incentives for them there again.

P9: The cost of the vaccination is high, it will prevent a lot of people from doing it. So for such an exercise, if it is not free, it should be subsidized to the maximum amount so that the ordinary person can afford it.

P5: i think effective follow-up is important. As we have mentioned earlier, being able to motivate staff to volunteer such as whenever the next vaccination is due, we can send people around the communities to either remind them or conduct this vaccination at the point of convenience to them, in their communities.

P10: I think lastly we can involve people of influence to add their voices too.

I: So we are targeting teenagers, girls especially. How can we establish a relationship with them so they can go for the vaccination? How will you address the situation? How will you arouse the interest enough to complete the vaccination?

P1: In our culture, it is very easy for adults to look down on teenagers maybe in a reprimanding thing. So in order to avoid that, you can win their trust by showing that you understand them instead of condemning them by telling them, why are you engaging in such an age; you try to get their views, understand them, get them on board. Once you gain their trust, they will come on board.

I: Right. Finally, what can the government, the metropolitan district or whatever do to promote and encourage vaccinations against HPV?

P2: As he said, the first thing you can do is to make it free or very subsidized.

P3: also to provide logistics that will be needed.

P4: They can also invocate these educational examples about the vaccines in their lessons so that the children are aware of the vaccines and cervical cancer.

I: In the absence of questions and comments, I would like to thank you for spending your time on this study.
